# Analysis of Cervical Spine Alignment and its Relationship with Other Spinopelvic Parameters after Laminoplasty in Patients with Degenerative Cervical Myelopathy

**DOI:** 10.3390/jcm9030713

**Published:** 2020-03-05

**Authors:** Seok Woo Kim, Seung Bo Jang, Hyung Min Lee, Jeong Hwan Lee, Min Uk Lee, Jeong Woo Kim, Jae Sung Yee

**Affiliations:** 1 Spine Center, Hallym University Sacred Heart Hospital, Hallym University, 896 Pyeongchon-dong, Dongan-gu, Anyang-si, Gyeonggi-do 431-070, Korea; ganzi404@naver.com (S.B.J.); lhm1011s@naver.com (H.M.L.); ljhsteve2338@gmail.com (J.H.L.); lmo932@hallym.or.kr (M.U.L.); hallymjw@naver.com (J.W.K.); id0917@naver.com (J.S.Y.); 2 Department of Orthopaedic Surgery, Hallym University Sacred Heart Hospital, Hallym University, 896 Pyeongchon-dong, Dongan-gu, Anyang-si, Gyeonggi-do 431-070, Korea

**Keywords:** cervical alignment, kyphosis, spinopelvic parameter, laminoplasty, myelopathy

## Abstract

For patients with kyphosis of the cervical spine, laminoplasty is usually incapable of improving neurological symptoms as it worsens kyphotic alignment. Thus, laminoplasty is not recommended in the presence of kyphotic alignment. Nevertheless, laminoplasty may be selected for myelopathy due to multiple-segment intervertebral disc herniation or ossification of posterior longitudinal ligament despite kyphotic alignment. This study examined whether cervical alignment influences surgical outcomes. Cervical alignment before the surgery was classified into lordosis and non-lordosis, and the non-lordosis group was subclassified into reducible and non-reducible groups to determine the change in cervical alignment before and after the surgery and to analyze its relationship with spinopelvic parameters. The lordosis group showed an increase in upper cervical motion (C0-2 Range of Motion (ROM), C0-2ROM/C0-7ROM) after surgery, while the non-lordosis group exhibited a decrease in C2-7ROM and C0-7ROM. The C0-2ROM was maintained without any reduction in the reducible group, while there was no significant change in cervical alignment and ROM of the non-reducible group. None of these changes showed significant association with the spinopelvic parameters of other sites. However, having a non-reducible type non-lordosis is not a proper indication for laminoplasty, as it does not change the alignment after surgery. Therefore, cervical alignment and reducibility should be identified before surgery.

## 1. Introduction

Laminoplasty is an effective surgical method that is commonly applied in patients with myelopathy due to multiple-segment ossification of the posterior longitudinal ligament, cervical herniated intervertebral disc, and spinal stenosis. This method is known to produce desired results to recover the blood flow of the decompressed spinal cord through nerve decompression via effective posterior migration of spinal cord after surgery if the lordotic alignment of the cervical spine is well maintained. On the other hand, the kyphotic curvature of cervical spine is not usually recommended as a good indication for laminoplasty in the group of patients who have kyphotic alignment of cervical spine because laminoplasty cannot achieve effective posterior migration of spinal cord after surgery, and it possibly worsens the kyphotic alignment [1,2,3,4,5,6,7]. However, laminoplasty may be inevitably selected for myelopathy due to multiple-segment intervertebral disc herniation or ossification of posterior longitudinal ligament despite kyphotic alignment.

According to the report [8], when radiological changes such as clinical outcomes, sagittal alignment, and overall range of motion (ROM) were compared after laminoplasty was conducted for the group of patients who had cervical kyphosis with Cobb angle <10°, there were no significant statistical differences from the cervical lordosis group. Furthermore, when multiple segments are involved for patients having kyphotic alignment of cervical spine, laminoplasty showed the same surgical outcome as for the patients with cervical lordosis within the range of Cobb angle <10°.

A recent study has examined the cervical alignment of 958 normal asymptomatic individuals, finding that the ratio of the subjects with kyphotic alignment of cervical spine comprises 26.3%. The group with kyphotic alignment can be subdivided into reducible kyphosis, in which the kyphotic curvature recovers to lordotic alignment in the lateral radiograph upon extension, and non-reducible kyphosis, in which the kyphotic alignment is sustained upon extension. According to this classification, among the 26.3% of subjects with kyphotic alignment, 15.7% (of the full 958-person cohort) was determined as non-reducible kyphosis [9]. 

In this regard, the authors classified the preoperative cervical alignment of the patients who underwent laminoplasty in our clinic into lordosis and non-lordosis (reducible vs. non-reducible) to analyze the relationship between the change in cervical alignment before and after surgery and its relationship with other spinopelvic parameters. The authors extend the analysis in determining whether the preoperative cervical alignment affects postoperative cervical alignment and other spinopelvic parameters, and, particularly, whether the group with preoperative kyphotic alignment of cervical spine shows a significant change in postoperative cervical alignment and other spinopelvic parameters. This study intends to confirm whether kyphotic alignment of cervical spine would be a proper indication for laminoplasty in terms of radiological analysis.

## 2. Materials and Methods

### 2.1. Research Subjects

**Figure 1:**
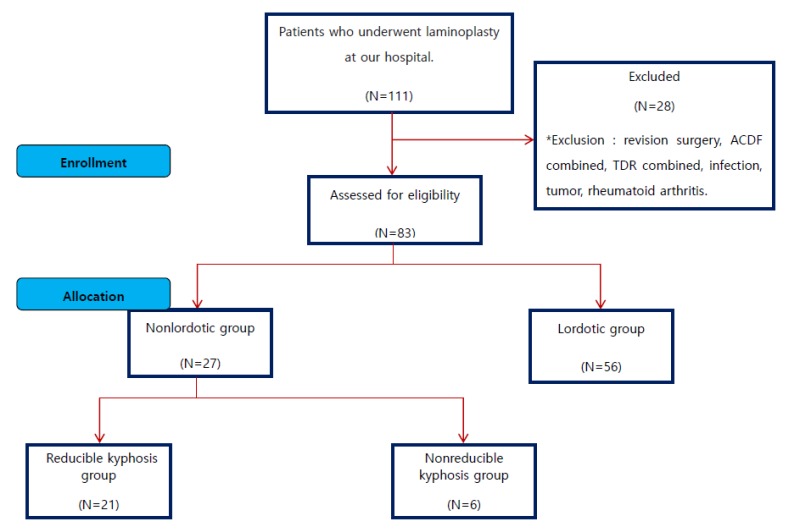
Flowchart of the study participants.

This study conducted a retrospective analysis by prospectively collecting data from 83 patients among 111 patients who underwent midline splitting double-door (French door) type laminoplasty based on a diagnosis of degenerative cervical myelopathy (DCM) from September 2008 to August 2015, excluding 28 patients with diagnosis of revision surgery, anterior cervical discectomy and fusion (ACDF) cases, total disc replacement (TDR) cases, infection, tumor, rheumatoid arthritis, etc. (Figure 1).

### 2.2. Surgical Technique

**Figure 2:**
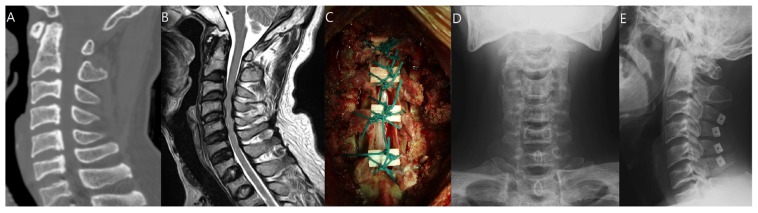
Pre-operative CT (**A**), pre-operative T2-weighted MRI (**B**), intra-operative photo (**C**), and post-operative AP (**D**) and lateral X-ray (**E**) of a 49-year-old male patient who underwent C3-6 midline splitting double-door type (French door type) laminoplasty.

A posterior incision was made along the nuchal ligament to the line of the spinous processes. The semispinalis cervicis was partially detached from the lower margin of the C2 spinous process. Cervical laminae were exposed laterally to the medial aspect of the facet joints, and the interspinous ligaments were removed. The involved spinous processes were split sagittally with a 0.54 mm diameter Tomita saw (T-saw; Medtronics, Memphis, TN, USA). It cut along the midline epidural space in a caudal-to-cranial direction. The advancing tip of the polyethylene sleeve was grasped when it appeared in the flavectomy at the other end of the decompression zone. The T-saw was advanced through the sleeve so that it could be held securely, whereas the sleeve was withdrawn retrograde over the saw. At this point, the unsheathed saw spanned the midline of the spinal canal along the area to be decompressed. Each end was grasped with a special clamp or needle holder. The T-saw was pulled tight before initiating a reciprocating motion. The T-saw should fit snugly just at the midline of the inner wall of the laminar arch. Continuous reciprocating motion cut the midline of the inner wall of the laminar arch and the spinous processes in a ventral-to-dorsal direction away from the dura and spinal cord. The supra- and interspinous ligaments were automatically dissected at the midline. The saw was frequently lubricated with sterile saline solution to avoid excessive heat and friction [10]. After bilateral gutters for the hinges were carefully made with a high-speed burr at the transitional area between the facet joint and the laminae, spinal canal enlargement was achieved by opening the split laminae bilaterally with a spreader and placing allo-bone graft (Laminar Spacer-K; CG Bio, Seoul, Korea; Figure 2).

### 2.3. Radiographic Measurement

This study used C-spine lateral X-rays, dynamic flexion–extension lateral radiography, and whole-spine lateral X-rays during the follow-up period before and after each surgery.

The radiographic protocol was standardized as follows. For each subject, cervical spine lateral radiographs were obtained with a 10 × 12 inch cassette at a 72 inch (182 cm) distance with the radiographic tube centered at the C4–C5 disc space with no magnification. The subjects were instructed to stand in a position with eyes looking forward and arms extended over their chests. Immediately after taking the cervical lateral neutral radiographs, flexion and extension views were obtained with a maximal neck flexion and extension position.

Whole-spine lateral radiographs were taken by using two 14 × 17 inch pieces of film in one 14 × 36 inch cassette at a 98.4 inch (250 cm) distance with the tube centered at the xiphoid process with no magnification. The subjects were instructed to stand in a position with eyes looking forward and arms crossed over their chests. The digital X-ray images were obtained and measured on a picture archiving and communication system (PiView, Infinitt, Seoul, Korea).

### 2.4. Radiological Analysis

**Figure 3:**
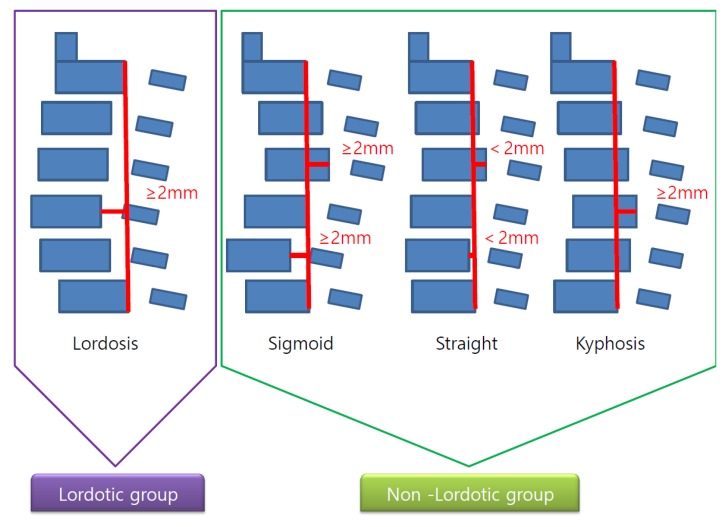
Diagram of lordosis and non-lordosis (kyphosis) groups.

**Figure 4:**
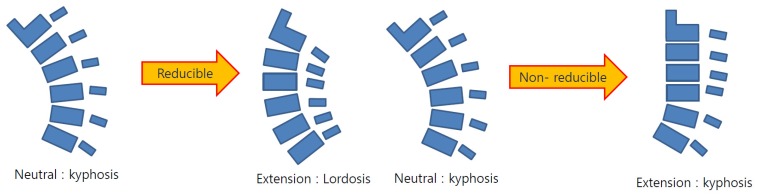
Diagram of reducible non-lordosis and non-reducible non-lordosis. Reducible, non-lordosis switches to lordosis in extension; non-reducible, non-lordosis (or kyphosis) maintains a non-lordotic (or kyphotic) alignment in extension.

C spine neutral X-ray was used to categorize the cervical alignment into lordotic and non-lordotic groups according to the Toyama classification [11], and the straight, sigmoid, and kyphosis groups were defined as a non-lordotic group (Figure 3). Afterwards, the analysis was conducted after subdividing the non-lordotic group into reducible non-lordotic group and non-reducible non-lordotic group, depending on the recovery of lordotic curve in the extension lateral X-ray view (Figure 4).

In each group, C2-7 angle, C0-2 angle, C0-7 angle, T1 slope, C0-2 ROM, C0-7 ROM, and C2-7 ROM were measured in the neutral, flexion, and extension views.

This study also evaluated spinopelvic parameters including TK (thoracic kyphosis), LL (lumbar lordosis), SS (sacral slope), PT (pelvic tilt), and PI (pelvic incidence) to identify the relationship between preoperative and postoperative cervical alignment and thoracolumbosacral alignment. The definitions of each of measurements are explained in Table 1.

### 2.5. Statistical Analysis

A *t*-test was used to analyze the measured parameters between the two groups, and ANOVA was utilized to analyze individual parameters among the three groups. Pearson correlation analysis was conducted to determine the correlation of the parameters among the groups. This study used the IBM SPSS software (version 22.0.0.1, IBM Corp., 2013, Armonk, NY, USA) in its statistical analysis. The statistical significance threshold was *p* < 0.05.

## 3. Results

The average age of all patients (59 males and 24 females) was 62.8, and the average follow-up period was 36.8 months. The lordosis group and the non-lordosis group included 56 (67.4%) and 27 (32.6%) patients, respectively; male to female ratios of 40:16 and 19:8, respectively; and average ages of 61.9 and 64.8, respectively (Table 2).

### 3.1. Post Operative Change of Curvature

The lordosis was maintained after surgery in 44 of the patients (78%) belonging to the lordosis group (*N* = 56) before the surgery. Although the lordosis was reduced in 12 patients (22%) after the surgery, only one patient exhibited no recovery of lordosis upon extension.

**Figure 5:**
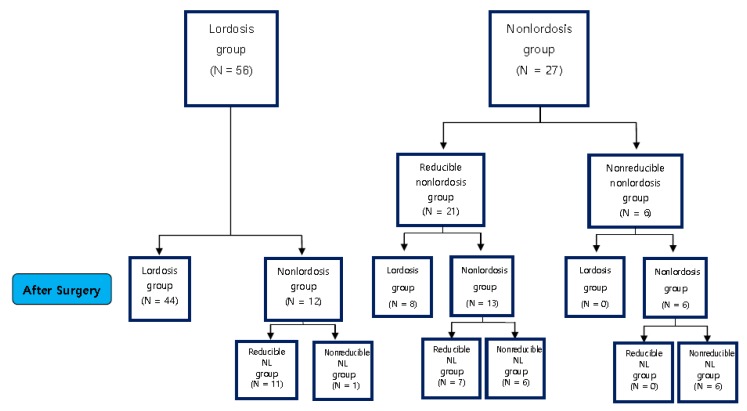
Post-operative change of curvature.

Of the patients belonging to the non-lordosis group (*N* = 27) before surgery, 21 (78%) fell under the reducible non-lordosis group showing the recovery of lordosis upon extension, and the remaining six (22%) fell under the non-reducible non-lordosis group exhibiting no recovery of lordosis upon extension. Eight patients (38%) in the reducible non-lordosis group (*N* = 21) changed into the lordosis group after the surgery, and seven (33%) and six (29%) shifted into the reducible non-lordosis group and the non-reducible non-lordosis group, respectively. All six patients belonging to the non-reducible non-lordosis group (*N* = 6) before surgery remained in the same group after the surgery (Figure 5).

#### 3.1.1. Comparison of Pre-Operative Radiological Parameters between the Lordosis and Non-Lordosis Groups

Among the parameters between the two groups, there were statistically significant differences in C0-2 angle (*p* < 0.01), C2-7 angle (*p* < 0.01), and the ratio of C0-2 ROM to C0-7 ROM (*p* = 0.05; Table 3). The reason why the ratio of C0-2 ROM to C0-7ROM in the non-lordosis group was high is that the more frequent use of upper cervical motion relatively compensates for the motion of the c-spine (C2-7 ROM). On the other hand, there were no statistically significant differences in other cervical and spinopelvic parameters (Table 3).

#### 3.1.2. Comparison of Post-Operative Radiological Parameters between the Lordosis and Non-Lordosis Groups

The overall cervical spine ROM, including C2-7 ROM and C0-7 ROM, decreased after surgery, while C0-2ROM increased, though not statistically significantly. The statistically significant differences among the parameters between the two groups were observed in C2-7 angle (*p* < 0.01), C0-2 angle (*p* = 0.02), C0-7 angle (*p* = 0.02), the ratio of C0-2 ROM to C0-7 ROM (C0-2/C0-7 ROM; *p* = 0.003), and T1 slope (*p* < 0.01). The reason why the ratio of C0-2 ROM to C0-7 ROM was high in the non-lordosis group is that the relative compensation of the upper cervical motion (C0-2 ROM) for the motion of C-spine (C2-7 ROM) is maintained after the surgery (Table 4).

The difference between the two groups was statistically significant for the C0-7 angle (*p* = 0.02) and T1 slope (*p* < 0.01) before and after the surgery, respectively. The lordosis of cervical spine reduction compared to before the surgery was statistically significantly different between the two groups (showing a greater decrease in the non-lordosis group). No statistically significant difference was observed in the other cervical and spinopelvic parameters (Table 4).

#### 3.1.3. Comparison of Pre-Operative vs. Post-Operative Radiological Parameters in the Lordosis Group

**Figure 6:**
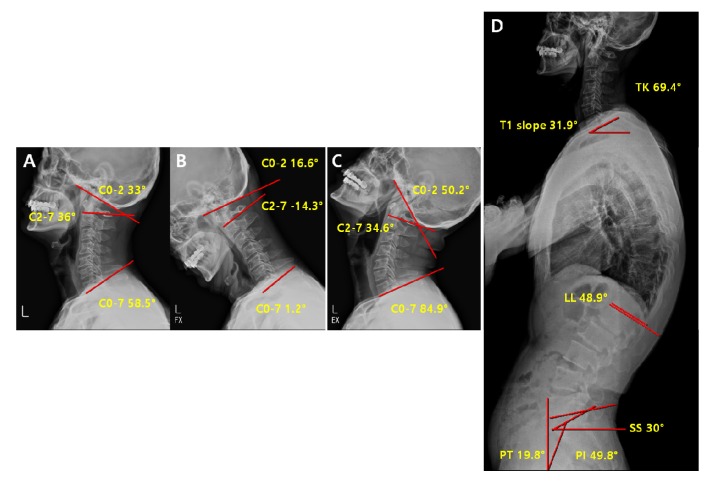
Pre-operative radiological parameters in lordosis groups. Cervical spine lateral X-ray of neutral (**A**), flexion (**B**), and extension (**C**), and whole-spine lateral X-ray (**D**). C0-2; C0-2 Cobb angle, the occipito-cervical angle, which is the intersection angle between the McGregor line and the line parallel to the C2 lower endplate. C2-7; C2-7 Cobb angle, the intersection angle between the line perpendicular to the line parallel to the C2 lower endplate and the line perpendicular to the line parallel to the C7 lower endplate. C0-7; C0-7 Cobb angle, the intersection angle between the McGregor line and the line parallel to the C7 lower endplate. TK; thoracic kyphosis, intersection angle between the line perpendicular to the line parallel to the T1 upper endplate and the line perpendicular to the line parallel to the T12 lower endplate. LL; lumbar lordosis, intersection angle between the line perpendicular to the line parallel to the L1 upper endplate and the line perpendicular to the line parallel to the L5 lower endplate. SS; sacral slope, the angle formed by a line drawn along the endplate of the sacrum and a horizontal reference line. PT; pelvic tilt, the angle formed by a line drawn from the midpoint of the sacral endplate to the center of the bicoxofemoral axis and a vertical and a vertical plumb line. PI; pelvic incidence, the angle formed by two vectors: the line joining the bicoxo-femoral axis to the center of the sacral end plate and the line perpendicular to the sacral endplate.

**Figure 7:**
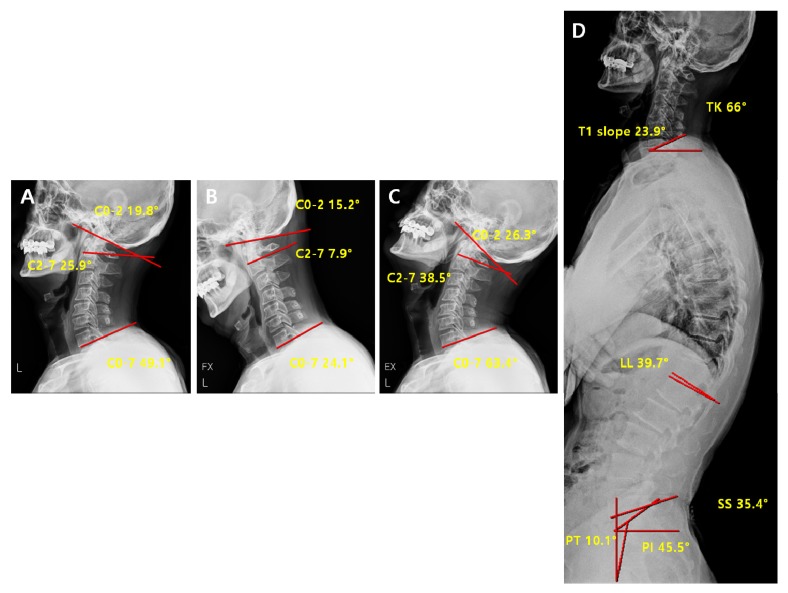
Post-operative radiological parameter in lordosis groups. Cervical spine lateral X-ray of neutral (**A**), flexion (**B**), extension (**C**) and whole-spine lateral X-ray (**D**).

Comparison of pre-operative vs. post-operative radiological parameters in the lordosis group is represented on Figure 6 and Figure 7.

In the lordosis group, the significant changes in the parameters before and after surgery were observed in C0-2 angle (*p* < 0.01), C0-7 angle (*p* = 0.03), C0-2ROM (*p* = 0.02), C2-7ROM (*p* < 0.01), C0-7ROM (*p* < 0.01), C0-2/C0-7 (*p* < 0.01), and C2-7/C0-7 (*p* < 0.01) (Table 5). After the surgery, the angle of c-spine (C0-7 angle) was maintained as lordotic, and C0-7ROM and C2-7ROM decreased, while C0-2 ROM and C0-2/C0-7ROM increased. These results suggest that the ROM of the cervical motion, which was reduced in the lordosis group after the surgery, was compensated through the upper cervical motion (C0-2). No statistically significant difference was observed in the other cervical and spinopelvic parameters (Table 5).

#### 3.1.4. Comparison of Pre-Operative vs. Post-Operative Radiological Parameters in the Non- Lordosis Group

In the non-lordosis group, the significant changes in the parameters before and after surgery were observed in C2-7ROM (*p* < 0.01), C0-7ROM (*p* < 0.01), and T1 slope (Table 6). Furthermore, as in the lordosis group, C2-7ROM and C0-7ROM decreased after the surgery, while C0-2ROM did not increase. These results suggest that the additional decrease in ROM after the surgery could not be compensated by the upper cervical spine (C0-2) because C0-2ROM was already heavily used before the surgery. In addition, change in T1 slope (decrease) was observed after the surgery, which means more kyphotic curvature (loss of lordosis) after surgery. No statistically significant difference was observed in the other cervical and spinopelvic parameters (Table 6).

#### 3.1.5. Comparison of Pre-Operative vs. Post-Operative Radiological Parameters in the Reducible Non-Lordosis Group

**Figure 8:**
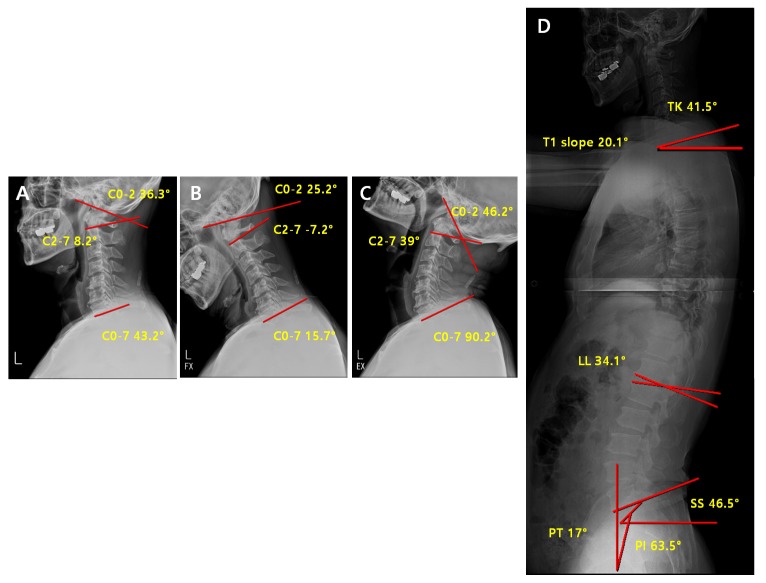
Pre-operative radiological parameters in the reducible non-lordosis group. Cervical spine lateral X-ray of neutral (**A**), flexion (**B**), extension (**C**) and whole-spine lateral X-ray (**D**).

**Figure 9:**
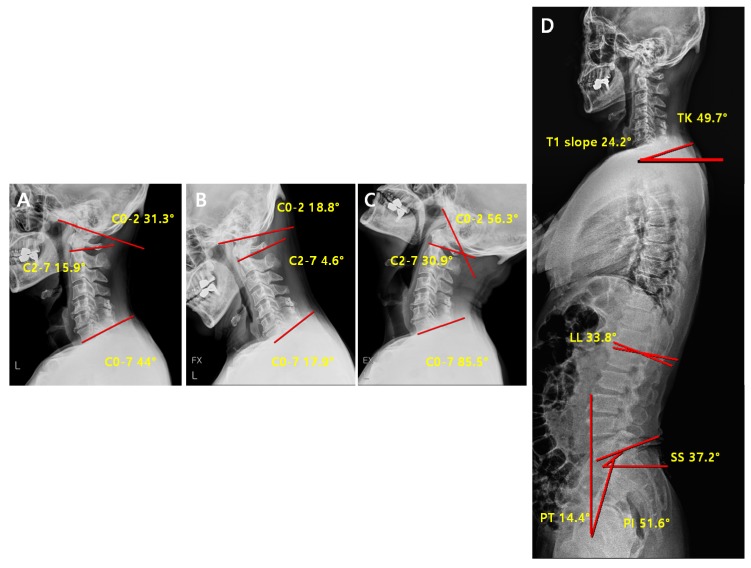
Post-operative radiological parameters in reducible non-lordosis group. Cervical spine lateral X-ray of neutral (**A**), flexion (**B**), extension (**C**) and whole-spine lateral X-ray (**D**).

In the reducible non-lordosis group, the significant changes in parameters before and after surgery were observed in C2-7 ROM (*p* < 0.01), C0-7 ROM *(p* < 0.01), and pelvic tilt (*p* = 0.02) (Table 7). However, no statistically significant difference was observed in the C0-2 ROM. These results suggest that the overall ROM decreases after the surgery, but not to an extent that requires compensation at the C0-2 site for maintaining the horizontal gaze or the ROM of cervical spine. No statistically significant differences were observed in the other cervical and spinopelvic parameters (Table 7). Comparison of pre-operative vs. post-operative radiological parameters in the reducible non-lordosis group is represented on Figure 8 and Figure 9

#### 3.1.6. Comparison of Pre-Operative vs. Post-Operative Radiological Parameters in the Non-Reducible Non-Lordosis Group

**Figure 10:**
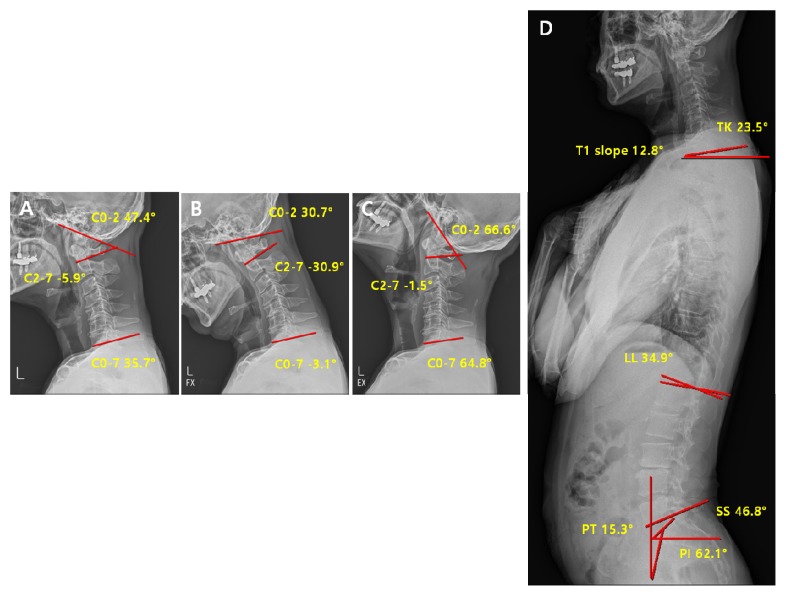
Pre-operative radiological parameters in the non-reducible non-lordosis group. Cervical spine lateral X-ray of neutral (**A**), flexion (**B**), extension (**C**) and whole-spine lateral X-ray (**D**).

**Figure 11:**
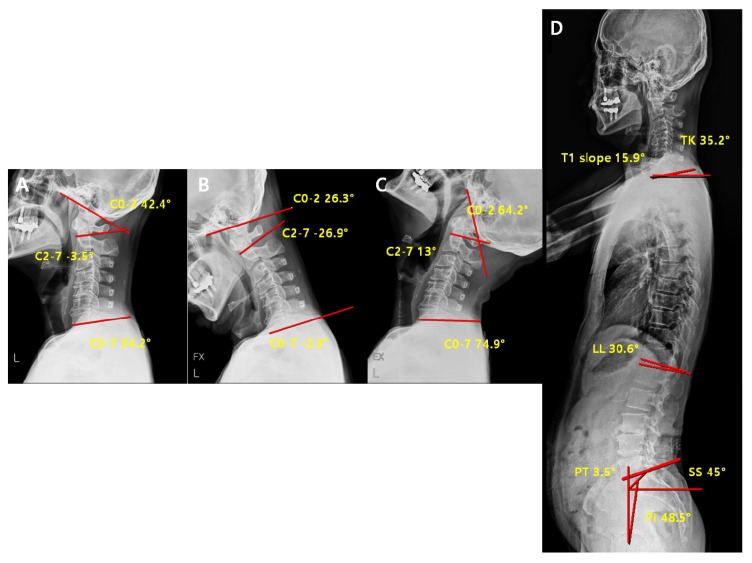
Post-operative radiological parameters in the non-reducible non-lordosis group. Cervical spine lateral X-ray of neutral (**A**), flexion (**B**), extension (**C**) and whole-spine lateral X-ray (**D**).

In the non-reducible non-lordosis group, no significant change in the c-spine parameters was observed before and after the surgery, and only the T1 slope decreased significantly (Table 8). These results suggest that no compensation for alignment or ROM before the surgery occurred at any sites after the surgery, and that the kyphosis of cervical spine was maintained after the surgery as the T1 slope related to the lordosis of cervical spine continues to significantly decrease. Thus, this group may not be a good indication for laminoplasty in comparison to the other groups because the application of laminoplasty does not change the kyphosis, hardly effective, in terms of all the credits from surgery including the decompression of neural tubes through posterior migration of spinal cord as well as the recovery of blood flow along the spinal cord. No statistically significant differences were observed in the other cervical and spinopelvic parameters (Table 8). Comparison of Pre-Operative vs. Post-Operative Radiological Parameters in the Non-Reducible Non-Lordosis Group (Figure 10 and Figure 11).

## 4. Discussion

Laminoplasty is an effective surgical method commonly applied in patients with myelopathy due to multiple-segment ossification of the posterior longitudinal ligament (OPLL) and spinal stenosis of cervical spine. In comparison to laminectomy and fusion surgery, laminoplasty is advantageous for DCM patients due to multiple OPLL or C-spine Herniated Nucleus Pulposus (HNP). The cervical motion can be maintained by preserving the posterior neck structure, such as the facet joint because neural tubes are extended on the laminofacet junction, where complications related to the use of instruments and direct adhesion between dura and overlying neck muscles can be reduced, and the recovery after surgery can be relatively prompt [1,4,5].

In general, it has been reported that laminoplasty is not effective for multiple-segment ossification of the posterior longitudinal ligament if kyphosis is present because indications for laminoplasty can benefit from decompression due to posterior migration of spinal cord only by maintaining the cervical lordosis [2,3,6,7,12].

However, studies have shown that kyphosis within 10° of cervical kyphosis has no different postoperative outcome from the cervical lordosis after laminoplasty [8]. Furthermore, a recent study has examined the cervical alignment of 958 normal asymptomatic individuals, finding that the ratio of the subjects with kyphotic alignment of cervical spine comprises 26.3%. The group with kyphotic alignment can be subdivided into reducible kyphosis, in which the kyphotic curvature recovers to lordotic alignment in the lateral radiograph upon extension, and non-reducible kyphosis, in which the kyphotic alignment is sustained upon extension. Although no distinctive cervical kyphosis features were observed between the two groups (reducible and nonreducible) in the resting neutral position, the feature of the motion between the two groups showed a difference in the flexion–extension ROM, depending on whether the upper cervical spine (C0–C2) or the lower cervical spine (C2–C7) ROM was compensated for the entire cervical motion. When the motion segments were divided into the upper cervical spine (C0–C2) and the lower cervical spine (C2–C7), each segment showed a distinctive ROM that served to preserve spinal functioning, especially horizontal gazing of the cervical spine. Also, this study identified that the correlation between the cervical spine and the global spine parameters (i.e., TK, LL, SS, PT, and PI) was not statistically significant, explaining the proper cervical spine alignment or ROM that moves the spine according to its relations with the head as the center is more necessary than focusing on the correlation between the cervical spine and the spinopelvic parameters [9].

In this regard, the authors conducted this study on the assumption that even if laminoplasty is performed in patients with cervical kyphosis, no kyphotic change is made, or the kyphotic change is minimized due to compensation of upper cervical motion (C0-2ROM) around the head center, and that the kyphosis where this upper cervical motion remains cannot be a contraindication for laminoplasty.

This study classified the curvature of the preoperative cervical spine into lordosis, reducible non-lordosis (kyphosis), and non-reducible non-lordosis (kyphosis) groups for the cases operated with cervical laminoplasty, respectively. Subsequently, this study examined the relationship between the cervical parameters and other spinopelvic parameters, and it further analyzed whether the characteristics of the curves changed after the surgery, and into which type the change would lead if it changed. Furthermore, this study analyzed whether the cervical parameter affected other spinopelvic parameters because these changes could determine whether cervical kyphosis would be a contraindication for laminoplasty in terms of radiological analysis.

The results of this study showed no difference in spinopelvic parameters between the lordotic group and the non-lordotic group before the surgery (Table 3). The results further indicated that the cervical curvature was independent of spinopelvic parameters regardless of curve characteristics of cervical spine. The results were consistent with those of other studies [8,13,14,15]. Moreover, the C2-7 angle before and after the surgery showed no statistically significant changes in both lordosis and non-lordosis groups (lordosis group *p* = 0.13, non-lordosis group *p* = 0.53).

There is one issue to discuss in this context. Many studies have typically measured the C2-7 angle in determining the overall ROM of the cervical spine. However, according to a recent study [9], in addition to the motion of C2-7, which is typically emphasized, the motion of C0-2 (upper cervical motion) plays an important role in the mobility of cervical spine. Thus, the authors have additionally measured C0-2 angle and ROM, C0-7 angle and ROM, as well as C2-7 angle and ROM to consider the upper cervical spine motion (C0-2) around the head center in this study.

The change before and after the surgery was examined by considering the above parameters in each group. The patients belonging to the lordosis group before the surgery showed no significant change in C2-7 angle after the surgery (*p* = 0.13), while exhibiting C0-7 angle changed to be more lordotic (between group difference mean=3.1, 95%CI: 1.3–5.4, *p*=0.03). C0-7ROM (between group difference mean= 9.2, 95%CI: 13.7 to 4.7, *p* < 0.01), and C2-7ROM (between group difference mean = 13.1, 95%CI: 16.4 to 9.8, *p* < 0.01) decreased while C0-2ROM increased (between group difference mean = 4.0, 95%CI: 0.8–7.2, *p* < 0.01). These results suggest that the ROM of cervical spine, which was reduced after the surgery, was compensated through the upper cervical spine motion (C0-2) around the head center. For this reason, the C0-2/C0-7 values also increased (between-group difference mean = −0.2, 95%CI: −0.2 to −0.1, *p* < 0.01) (Table 5).

Furthermore, according to the change in the postoperative curvature, only one of the patients belonging to the lordosis group before the surgery changed to the non-reducible non-lordosis group after the surgery, and the remaining patients maintained lordosis after surgery or its recovery upon extension even after the loss of lordosis (Figure 5).

Thus, in the patients with the lordotic curve of cervical spine before the surgery, the lordosis was mostly maintained or compensated after the surgery, and the overall ROM of the cervical spine (C2-7, C0-7 ROM) was inevitably reduced due to posterior approach surgery, while the upper cervical spine motion (C0-2 ROM, C0-2/0-7 ROM) acted in a compensatory way around the head center, resulting in maintenance of horizontal gaze and daily activities after the surgery. Accordingly, the results suggest that lordosis can be a good indication for the surgery because the effective decompression and posterior migration of the spinal cord after the surgery would recover the blood flow.

On the other hand, the patients belonging to the non-lordosis (kyphosis) group before the surgery showed a decrease in C2-7ROM and C0-7ROM after the surgery, as in the lordosis group, while exhibiting no increase in C0-2ROM unlike the lordosis group (Table 6). This suggests that there was a limit or inability to compensate for the additional decrease of ROM with the upper cervical motion (C0-2 ROM) because the C0-2ROM was already much used before the surgery. Unlike the lordosis group, the T1 slope, which indicates the lordosis of cervical spine, also tended to decrease after the surgery.

The non-lordosis (kyphosis) group was divided into reducible and non-reducible groups according to the recovery of lordosis upon extension of cervical spine to analyze the change in parameters before and after the surgery: in the reducible non-lordosis group, C2-7ROM (*p* < 0.01) and C0-7ROM (*p* < 0.01) significantly decreased, while C0-2 ROM remained unchanged. However, in the non-reducible non-lordosis group, only the T1 slope showed a significant decrease, indicating that the kyphotic state proceeded after the surgery; no changes in other parameters were observed. The change in cervical parameters, whether it was increase or decrease, was observed in all the groups other than the non-reducible non-lordosis group, while all the values including cervical spine angle and ROM were maintained after the surgery only in the non-reducible non-lordosis group. The results suggest no variability in cervical motion and angle after the surgery unlike other groups. In other words, in the non-reducible non-lordosis group, the spine move in the state C0-2, C2-7, C0-7, angle and ROM are already fixed around the head center before surgery, and the ROM of cervical spine remains unchanged without any improvement after surgery, which is not compensated by the upper cervical motion (C0-2) after the surgery.

Furthermore, the curvature changes after surgery in the non-lordosis (or kyphosis) group showed that 8 (38%) of 21 patients who belonged to the reducible non-lordosis group changed to the lordosis group, 7 (33%) remained in the reducible non-lordosis group, and 6 (29%) changed into the non-reducible non-lordosis group. On the other hand, all six patients in the non-reducible non-lordosis group remained in the same group after the surgery.

Based on the above results, non-reducible characteristics remain unchanged after the surgery in the non-reducible non-lordosis group (that is, the characteristics in which lordosis does not recover during extension), showing no variability in the cervical angle and ROM, and no compensation through the upper cervical motion (C0-2 ROM). Thus, no effect of laminoplasty (improvement effect of blood flow in the spinal cord due to the posterior migration of the spinal cord) would be observable, while the preoperative characteristics remain unchanged after the surgery. On the other hand, the reducible non-lordosis group and the lordosis group mostly show variability, and the upper cervical motion (C0-2 ROM) around the head center acts in a compensatory way. Accordingly, patients who have lordosis or reducible non-lordosis can be a good indication for laminoplasty by considering these quantitative and qualitative changes in curve, angle, and ROM of cervical spine before and after the surgery, which are independent of other spinopelvic parameters. However, selective surgery is required because some limited cases among them show characteristics that are not reducible after the surgery, and further studies are necessary to determine the selection method.

### Limitations

This study has the following limitations. Firstly, this study is a retrospective study of prospectively collecting data, and despite a relatively long-term observation (36.8 months on average), 28 of 111 patients were lost during the follow-up period (25%). Furthermore, the number of the entire patient group was 83, which was not small, while the entire group was divided into subgroups, and the number of each group was relatively small to show statistical significance (non-reducible non-lordosis: 6 patients). This limitation, including a small number of patients in this study, arose because laminoplasty typically involves the lordosis of the cervical spine, and laminoplasty is rarely performed for patients with kyphosis, except in special cases. Secondly, all patients included in this study underwent midline splitting double-door laminoplasty (French door type laminoplasty). For this reason, these postoperative results could differ from those of laminoplasty applying different surgical methods (e.g., unilateral expansive open door type laminoplasty). Finally, this study has radiologically analyzed how the preoperative curve of cervical spine would change after surgery, and whether the changed curve would affect cervical parameters and other spinopelvic parameters, which could be independent of clinical results of patients before and after the surgery.

## 5. Conclusions

Considering radiographic changes in curve, angle, and ROM of cervical spine and its relationship with other spinopelvic parameters before and after the surgery, only patients with cervical lordosis or reducible kyphosis should be considered for laminoplasty surgery.

## Figures and Tables

**Table 1:** Definitions of measurements.

Parameters	Definition
C2-7 Cobb angle	The intersection angle between the line perpendicular to the line parallel to the C2 lower endplate and the line perpendicular to the line parallel to the C7 lower endplate
C0-2 Cobb angle	The occipito-cervical angle, which is the intersection angle between the McGregor line and the line parallel to the C2 lower endplate and is used to evaluate the curvature of the upper cervical spine
C0-7 Cobb angle	The intersection angle between the McGregor line and the line parallel to the C7 lower endplate
C0-2/C0-7	The value of C0-2 Cobb angle divided by C0-7 Cobb angle
C2-7/C0-7	The value of C2-7 Cobb angle divided by C0-7 Cobb angle
T1 slope	The intersection angle between the tangent line and the upper plate of the T1 vertebral body
SS (Sacral Slope)	The angle formed by a line drawn along the endplate of the sacrum and a horizontal reference line
PT (Pelvic Tilt)	The angle formed by a line drawn from the midpoint of the sacral endplate to the center of the bicoxofemoral axis and a vertical and a vertical plumb line
PI (Pelvic incidence)	The angle formed by two vectors: (1) The line joining the bicoxo-femoral axis to the center of the sacral end plate and (2) A line perpendicular to the sacral endplate

**Table 2:** Demographic data of the study participants.

Patient Who Underwent Laminoplasty (*n* = 83).			
	Lordosis	Non-Lordosis	*p*-Value *
No. of participants	56 (67.4%)	27 (32.6%)	
Sex ratio (M:F)	40:16	19:8	0.921
Age [95% CI]	61.9 [60.4~63.4]	64.8 [62.6~67.0]	0.292

CI, confidence interval. Chi-square test for sex ratio, independent t test for age. * Statistical significance (*p* < 0.05).

**Table 3:** Comparison of pre-operative radiographic parameters between lordosis and non-lordosis.

Parameter	Lordosis Mean (Degrees)	Non-Lordosis Mean (Degrees)	Between-Group Difference Mean (Degrees) (95% CI)	*p*-Value
C2-7angle	13.2(10.9~15.9)	1.1(−2.6~4.9)	12.1(7.7~16.5)	0.01 *
C0-2angle	24.2(21.2~26.8)	31.9(27.9~35.9)	−7.7 (−12.6~-2.8)	0.01 *
C0-7angle	36.8(33.3~40.4)	33.4(29.6~37.3)	3.4 (−2.4~9.1)	0.25
C0-2ROM	17.8(15.3~20.1)	23.1(16.2~30.1)	−5.3(−11.1~0.5)	0.07
C2-7ROM	34.3(30.7~37.6)	29.7(24.9~34.5)	4.6(−1.3~10.5)	0.12
C0-7ROM	52.4(47.7~56.4)	50.1(44.9~55.3)	2.3(−4.9~9.5)	0.53
C0-2/C0-7	0.3(0.3~0.4)	0.5(0.3~0.7)	−0.1(−0.3~0)	0.05 *
C2-7/C0-7	0.7(0.6~0.7)	0.6(0.5~0.7)	0.1(0~0.2)	0.18
T1slope	29.7(27.2~32.4)	26.9(23.5~30.3)	2.8(−1.6~7.2)	0.21
Lumbar lordosis	37.6(25.9~49.3)	26.4(12.3~39.9)	11.2(−0.8~23.3)	0.07
sacral slope	34.0(29.6~38.5)	30.9(21.7~40.1)	3.1(−5.9~12.1)	0.49
pelvic tilt	15.5(12.1~18.8)	16.5(9.6~23.4)	−1.0(−7.8~5.8)	0.76
pelvic incidence	47.4(40.8~53.9)	41.6 (23.8~50.5)	5.7 (−6.6~18.1)	0.35

CI, confidence interval; ROM, range of motion * Statistical significance (*p* < 0.05).

**Table 4:** Comparison of post-operative radiographic parameters between lordosis and non-lordosis.

Parameter	Lordosis Mean (Degrees)	Non-Lordosis Mean (Degrees)	Between-Group Difference Mean (Degrees) (95% CI)	*p*-Value
C2-7angle	11.3(8.4~14.4)	−0.1(-4.9~4.8)	11.4(5.9~16.9)	0.01 *
C0-2angle	27.2(24.2~30.5)	33.7(29.8~37.7)	−6.5(−11.8~-1.3)	0.02 *
C0-7angle	39.7(36.1~43.7)	32.9(29.5~36.3)	6.9(1.0~12.8)	0.02 *
C0-2ROM	21.7(18.6~24.7)	24.8(20.9~28.7)	−3.1(−8.2~2.0)	0.23
C2-7ROM	20.9(18.1~23.9)	19.8(14.6~24.9)	1.2(−4.2~6.6)	0.66
C0-7ROM	42.8(39.2~46.6)	40.9(35.2~46.7))	1.9(−4.7~8.5)	0.57
C0-2/C0-7	0.5(0.4~0.6)	0.6(0.5~0.7)	−0.1(−0.2~0)	0.03 *
C2-7/C0-7	0.5(0.4~0.6)	0.5(0.4~0.6)	0.0(−0.1~0.1)	0.85
T1slope	29.3(26.9~32.3)	23.3(20.3~26.3)	6.0(1.7~10.3)	0.01 *
Lumbar lordosis	32.7(21.2~44.2)	36.2(27.8~44.6)	−3.5(−20.2~13.2)	0.64
sacral slope	36.0(32.6~43.8)	39.1(31.0~47.2)	−3.0(−7.7~1.6)	0.17
pelvic tilt	19.0(12.4~28.0)	20.1(-2.7~42.9)	−1.1(−16.0~13.8)	0.87
pelvic incidence	55.0(48.3~68.3)	55.6(35.4~75.7)	−0.5(−13.4~12.3)	0.93

CI, confidence interval; ROM, range of motion * Statistical significance (*p* < 0.05)

**Table 5:** Comparison of pre-operative vs. post-operative radiological parameters of lordosis groups.

Parameter	Lordosis			
	Pre-op	Post-op	Between-Group Difference Mean (Degrees) (95% CI)	*p*-Value
	Mean (Degrees)	Mean (Degrees)		
C2-7angle	13.2(10.9~15.9)	11.3(8.4~14.4)	2.0(0.6~4.6)	0.13
C0-2angle	24.2(21.2~26.8)	27.2(24.2~30.5)	−3.4(−5.4~−1.3)	<0.01 *
C0-7angle	36.8(33.3~40.4)	39.7(36.1~43.7)	−3.1(−5.9~−0.3)	0.03 *
C0-2ROM	17.8(15.3~20.1)	21.7(18.6~24.7)	−4.0(−7.2~−0.8)	0.02 *
C2-7ROM	34.3(30.7~37.6)	20.9(18.1~23.9)	13.1(9.8~16.4)	<0.01 *
C0-7ROM	52.4(47.7~56.4)	42.8(39.2~46.6)	9.2(4.7~13.7)	<0.01 *
C0-2/C0-7	0.3(0.3~0.4)	0.5(0.4~0.6)	−0.2(−0.2~−0.1)	<0.01 *
C2-7/C0-7	0.7(0.6~0.7)	0.5(0.4~0.6)	0.2(0.1~0.2)	<0.01 *
T1slope	29.7(27.2~32.4)	29.3(26.9~32.3)	0.2(-2.1~2.4)	0.89
Lumbar lordosis	37.6(25.9~49.3)	32.7(21.2~44.2)	−1.1(−6.4~4.3)	0.57
sacral slope	34.0(29.6~38.5)	36.0(32.6~43.8)	−0.7(−10.8~9.5)	0.85
pelvic tilt	15.5(12.1~18.8)	19.0(12.4~28.0)	1.2(−10.1~12.4)	0.77
pelvic incidence	47.4(40.8~53.9)	55.0(48.3~68.3)	0.8(−14.0~15.6)	0.88

CI, confidence interval; ROM, range of motion. * Statistical significance (*p* < 0.05).

**Table 6:** Comparison of pre-operative vs. post-operative radiological parameters of non-lordosis groups.

Parameter	Non-Lordosis			
	Pre-op	Post-op	Between-Group Difference MEAN (Degrees) (95% CI)	*p*-Value
	Mean (Degrees)	Mean (Degrees)		
C2-7angle	1.1(-2.6~4.9)	−0.1(−4.9~4.8)	1.2(2.6~5.0)	0.53
C0-2angle	31.9(27.9~35.9)	33.7(29.8~37.7)	−1.9(−5.1~1.4)	0.26
C0-7angle	33.4(29.6~37.3)	32.9(29.5~36.3)	0.6(−3.6~4.7)	0.78
C0-2ROM	23.1(16.2~30.1)	24.8(20.9~28.7)	−1.7(−9.5~6.2)	0.67
C2-7ROM	29.7(24.9~34.5)	19.8(14.6~24.9)	9.9(4.5~15.4)	<0.01 *
C0-7ROM	50.1(44.9~55.3)	40.9(35.2~46.7)	9.2(2.6~15.8)	<0.01 *
C0-2/C0-7	0.5(0.3~0.7)	0.6(0.5~0.7)	−0.1(−0.3~0.1)	0.14
C2-7/C0-7	0.6(0.5~0.7)	0.5(0.4~0.6)	0.1(−0.02~0.2)	0.1
T1slope	26.9(23.5~30.3)	23.3(20.3~26.3)	3.6(0.1~7.0)	0.04 *
Lumbar lordosis	26.4(12.3~39.9)	36.2(27.8~44.6)	2.4(−4.0~8.9)	0.35
sacral slope	30.9(21.7~40.1)	39.1(31.0~47.2)	−0.7(−4.3~3.0)	0.64
pelvic tilt	16.5(9.6~23.4)	20.1(−2.7~42.9)	−0.9(−13.6~11.8)	0.85
pelvic incidence	41.6 (23.8~50.5)	55.6(35.4~75.7)	−4.1(−18.9~10.7)	0.49

CI, confidence interval; ROM, range of motion * Statistical significance (*p* < 0.05).

**Table 7:** Comparison of pre-operative vs. post-operative radiological parameters of the reducible non-lordosis group.

Parameter	Reducible Non-Lordosis			
	Pre-op	Post-op	Between-Group Difference Mean (Degrees) (95% CI)	*p*-Value
	Mean (Degrees)	Mean (Degrees)		
C2-7angle	2.0(−2.3~6.2)	2.5(−2.9~7.9)	−0.5(−4.8~3.8)	0.81
C0-2angle	29.9(25.5~34.4)	31.5(27.6~35.5)	−1.6(−5.6~2.4)	0.42
C0-7angle	32.2(27.7~36.6)	33.3(29.2~37.4)	−1.1(−6.1~3.8)	0.64
C0-2ROM	23.7(−14.8~32.5)	23.6(19.4~27.9)	0.03(−10.0~10.0)	0.99
C2-7ROM	30.3(24.2~36.4)	18.7(12.3~25.2)	11.6(5.2~18.0)	<0.01 *
C0-7ROM	51.5(45.1~57.9)	37.9(32.0~43.8)	13.6(6.7~20.5)	<0.01 *
C0-2/C0-7	0.5(0.2~0.7)	0.6(0.5~0.8)	−0.2(−0.4~0.1)	0.20
C2-7/C0-7	0.6(0.5~0.7)	0.5(0.3~0.7)	−0.2(−0.4~0.1)	0.27
T1slope	26.3(22.1~30.5)	25.6(22.6~28.6)	0.7(2.5~3.9)	0.67
Lumbar lordosis	37.0(35.0~39.0)	32.4(25.4~39.4)	4.7(76.0~85.3)	0.60
sacral slope	40.9(36.5~47.3)	40.8(38.7~42.9)	0.1(−39.3~39.5)	0.98
pelvic tilt	13.3(8.1~18.5)	15.3(10.1~20.5)	−2.0(−2.6~-1.3)	0.02 *
pelvic incidence	54.8(44.5~65.1)	50.9(49.9~51.9)	3.9(−79.4~87.1)	0.66

CI, confidence interval; ROM, range of motion * Statistical significance (*p* < 0.05).

**Table 8:** Comparison of pre-operative vs. post-operative radiological parameters of the non-reducible non-lordosis group.

Parameter	Nonreducible Non-Lordosis			
	Pre-op	Post-op	Between-Group Difference Mean (Degrees) (95% CI)	*p*-Value
	Mean (Degrees)	Mean (Degrees)		
C2-7angle	−1.8(−12.5~8.8)	−9.0(−19.5~−1.6)	7.1(−1.8~16.0)	0.10
C0-2angle	38.7(29.7~47.8)	41.5(30.0~53.0)	−2.8(−10.1~4.6)	0.37
C0-7angle	37.9(28.9~47.0)	31.5(24.3~38.6)	6.5(1.5~14.4)	0.09
C0-2ROM	21.4(12.2~30.5)	28.9(16.6~41.3)	−7.6(−16.4~1.3)	0.08
C2-7ROM	27.5(20.6~34.5)	23.3(15.2~31.4)	4.2(−8.5~16.9)	0.43
C0-7ROM	45.1(36.0~54.2)	51.4(34.7~68.1)	−6.3(−19.7~7.1)	0.28
C0-2/C0-7	0.5 (0.3~0.7)	0.5(0.4~0.7)	−0.1(−0.2~0.1)	0.24
C2-7/C0-7	0.6(0.4~0.8)	0.5(0.4~0.5)	0.2(0.0~0.4)	0.09
T1slope	28.8(22.9~34.7)	15.2(9.7~20.6)	13.7(7.1~20.2)	<0.01 *
Lumbar lordosis	31.3(16.2~46.4)	28.0(18.1~37.9)	3.0(−4.1~10.1)	0.28
sacral slope	33.4(22.2~44.6)	35.0(26.9~43.1)	−0.4(−4.5~3.8)	0.79
pelvic tilt	12.8(5.0~20.6)	15.0(9.2~20.8)	−2.8(−23.4~17.7)	0.69
pelvic incidence	46.1(33.1~59.1)	41.7(20.7~62.7)	−3.2(−25.1~18.7)	0.67

CI, confidence interval; ROM, range of motion. * Statistical significance (*p* < 0.05).
